# Sugar Metabolism and Transcriptome Analysis Reveal Key Sugar Transporters during *Camellia oleifera* Fruit Development

**DOI:** 10.3390/ijms23020822

**Published:** 2022-01-13

**Authors:** Yu He, Ruifan Chen, Ying Yang, Guichan Liang, Heng Zhang, Xiaomei Deng, Ruchun Xi

**Affiliations:** 1 Department of Forestry, College of Forestry and Landscape Architecture, South China Agricultural University, Guangzhou 510642, China; yuh@stu.scau.edu.cn (Y.H.); 20202159004@stu.scau.edu.cn (R.C.); heng@stu.scau.edu.cn (Y.Y.); twocicada@163.com (G.L.); ginkgozh@163.com (H.Z.); 2 Guangdong Key Laboratory for Innovative Development and Utilization of Forest Plant Germplasm, Guangzhou 510642, China

**Keywords:** *Camellia oleifera*, fruit development, sugar content, enzymes in sucrose metabolism, RNA-seq, sucrose transport

## Abstract

*Camellia oleifera* is a widely planted woody oil crop with economic significance because it does not occupy cultivated land. The sugar-derived acetyl-CoA is the basic building block in fatty acid synthesis and oil synthesis in *C. oleifera* fruit; however, sugar metabolism in this species is uncharacterized. Herein, the changes in sugar content and metabolic enzyme activity and the transcriptomic changes during *C. oleifera* fruit development were determined in four developmental stages (CR6: young fruit formation; CR7: expansion; CR9: oil transformation; CR10: ripening). CR7 was the key period of sugar metabolism since it had the highest amount of soluble sugar, sucrose, and glucose with a high expression of genes related to sugar transport (four sucrose transporters (SUTs) or and one SWEET-like gene, also known as a sugar, will eventually be exported transporters) and metabolism. The significant positive correlation between their expression and sucrose content suggests that they may be the key genes responsible for sucrose transport and content maintenance. Significantly differentially expressed genes enriched in the starch and sucrose metabolism pathway were observed in the CR6 versus CR10 stages according to KEGG annotation. The 26 enriched candidate genes related to sucrose metabolism provide a molecular basis for further sugar metabolism studies in *C. oleifera* fruit.

## 1. Introduction

Photosynthesis is the process by which green plants absorb sunlight, assimilate CO_2_ and H_2_O, produce a photosynthate, and release O_2_. Sucrose is the main photosynthate which serves as the basis for the synthesis of carbon and the storage of protein, starch, and lipids for plant growth [1,2]. It is exported from leaves (source tissues) to different non-photosynthetic plant organs (sink tissues). Photosynthates and assimilates are transported through the vascular system and distributed to various organs. The process is divided into two stages: loading and unloading. Loading involves photosynthate transport from mesophyll cells to the sieve element–companion cell (SE–CC) complex. Unloading involves exporting photosynthates from the phloem to adjacent growth sites or storage tissues. Plants have an adaptive mechanism that actively regulates photosynthate distribution to adapt to different physiological needs and environmental stresses [3]. This distribution and transport affects plant growth and development based on its source strength and storage capacity [4]. When sucrose production by photosynthesis and transport efficiency from the source (leaf) to the sink (organ) increases, the economic yield of crops increases and a higher economic value is obtained [5]. Overexpression of the potato sucrose symporter *StSUT1* in the parenchyma of developing pea seed sink cells increases cotyledon growth rate through the sucrose influx [6]. Therefore, upregulating sucrose transporter expression is an effective means to regulate source–sink allocation to increase crop economic yield [7]. High expression of the plant sugar transport-related protein VvSWEET15 increases hexose sugar accumulation in *Vitis vinifera* L. [8,9]. Similarly, AtSWEET17 in *Arabidopsis thaliana* controls the fructose content in leaves [10]. Meanwhile, the *MdSUT4.1* expression level of the sucrose transporter in *Malus* x *domestica* negatively correlates with fruit sugar content, indicating that it transports sugar out of vacuoles [11], and BnSUT1C mediates sucrose absorption by pollen in the late stage of anther development in *Brassica napus* L. [12]. The photo-assimilates from the source organs enter the fruit through the phloem in the form of sucrose. The strict regulation of sucrose synthesis, transport and use affects fruit growth and development [13]. In recent years, sucrose metabolism has been extensively studied in such edible fruit as *Prunus persica* [14], *Prunus armeniaca* [15,16], grapes [8], apples [11,17], and *Sacharum officinarum* [18], plus *Allium cepa* [19], *Beta vulgaris* [20], and *Arachis hypogaea* [21]. Sucrose synthase (SuSy), sucrose phosphate synthase (SPS), neutral invertase (NI), cell wall acid invertase (CWINV), and soluble acid invertase (SAI) significantly affect the synthesis and catabolism of soluble sugars in fruit [8,22,23].

*Camellia oleifera* Abel. is a woody oil-tea tree belonging to the Theaceae family, which is endemic in China and has a planting history of more than 2300 years [24,25]. It is one of the world’s four major woody edible oil plants together with oil palm, olive, and coconut. Oil tea seeds produce a high-grade edible oil that prevents cardiovascular disease and scavenges free radicals, and they have an unsaturated fatty acid content above 90% [26,27]. It is favored by consumers and is in great demand. There are no related studies on the accumulation and transport of *C. oleifera* photosynthate and previous studies of photosynthate distribution in other species have mostly focused on the allocation characteristics of long-term accumulation [28,29]. The distribution of photosynthates in *Fagus sylvatica* seedlings is fine root > thick root > leaf > stem > twig using ^13^C isotope pulse labeling [30], while the distribution of newly synthesized photosynthates in *Triticum aestivum* is significantly higher in aboveground than in underground tissues [31]. Therefore, studying the transport and distribution characteristics of assimilates in different *C. oleifera* developmental stages by ^13^C labeling provides a basis for improving *C. oleifera* economic yield. Sucrose metabolism regulation in *C. oleifera* fruit is not known although many genes involved in sucrose transport and metabolism are reported in other species. The identification of important sucrose transport and metabolism-related genes during fruit development is important for oil synthesis in late development of *C. oleifera* since sucrose is a prerequisite for oil synthesis [32].

In this study, ^13^C pulse labeling was used to determine and analyze the accumulation and distribution of photosynthates and the sugar content and sucrose metabolism related enzyme activities in four developmental stages of *C. oleifera* fruits were selected as the research objects: CR6: young fruit formation stage; CR7: fruit expansion stage; CR9: fruit oil transformation stage; CR10: fruit ripening stage. In addition, candidate genes were identified with important roles in sucrose accumulation and metabolism. A theoretical basis for the source and sink regulation in *C. oleifera* was determined, which provided technical support for improving its yield and quality.

## 2. Results

### 2.1. Isotopic Abundance Changes of Different Organs at Different Developmental Stages

**Figure 1:**
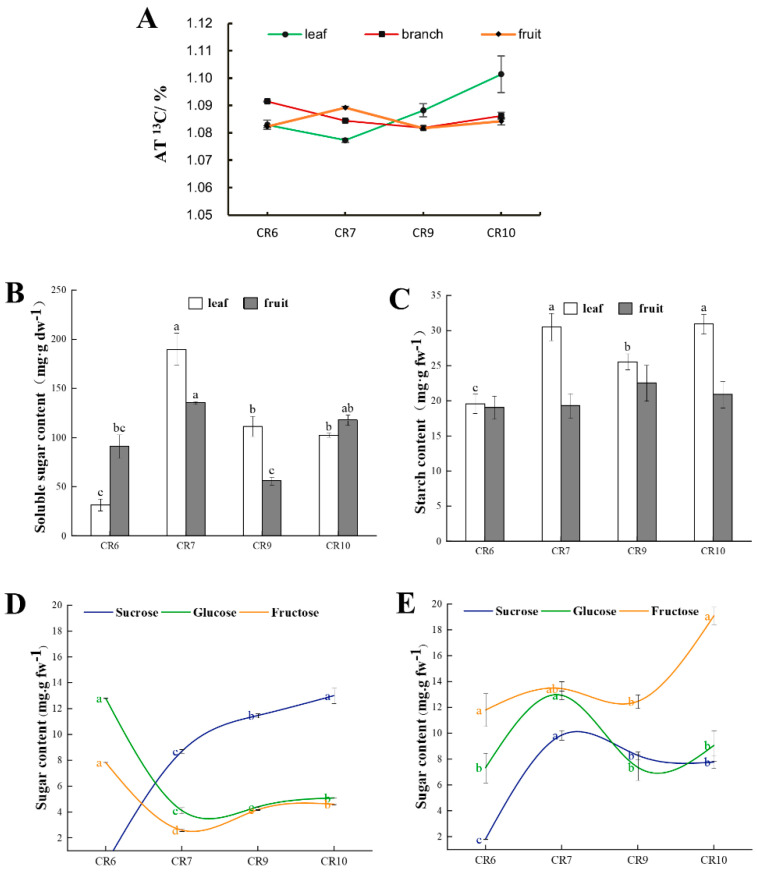
^13^C abundance, starch and sugar content of different *Camellia oleifera* organs at four developmental stages: CR6, young fruit formation; CR7, fruit expansion; CR9, fruit oil transformation; CR10, fruit ripening. (**A**) The abundance of ^13^C in leaves, branches and fruits was measured 72 h after ^13^C labeling. The *y*−axis represents the atomic percentage. (**B**) Soluble sugar content in leaves and fruit. (**C**) Starch content in leaves and fruit. (**D**) Sucrose, glucose and fructose content in leaf. The sucrose content is counted as 0 in CR6 in leaves because it is below the limit of detection by high performance liquid chromatography (HPLC). (**E**) Sucrose, glucose and fructose content in fruit. Significant differences (*p* < 0.05) are indicated by lower case letters which are arranged alphabetically starting from the highest values. Similar letters indicates no significant difference. All error bars depict the SD of the mean (n = 3).

The ^13^C abundance of labeled leaves decreased from CR6 to CR7 then increased and reached a maximum value (1.1014%) in CR10 (Figure 1A). The ^13^C abundance of labeled branches slightly decreased from 1.0915% in CR6 to 1.0818% in CR9 then slightly increased to 1.0862% in CR10. The ^13^C abundance of labeled fruit followed a gentle fluctuating trend where it initially increased, then decreased, and increased again. Its ^13^C abundance reached a maximum at CR7 (1.0892%) while the minimum was 1.0823% in CR6.

### 2.2. Soluble Sugar and Starch Concentration during Fruit Development

The soluble sugar content in *C. oleifera* leaves was the lowest at CR6 (31.8 mg g^−1^) with a maximum value of 212.5 mg g^−1^ in CR7, followed by a decrease to 111.6 mg g^−1^ in CR9 and 102.4 mg g^−1^ in CR10. The soluble sugar content in the fruit initially increased to a maximum value of 135.6 mg g^−1^ at CR7, then decreased to a minimum value of 56 mg g^−1^ at CR9, and increased again to 117.8 mg g^−1^ in CR10 (Figure 1B). The fruit sucrose content was the lowest in at CR6 (1.81 mg g^−1^) but rose to 8.32 mg g^−1^ in in CR7. The content did not change significantly between CR9 and CR10, which showed an overall increasing trend. Glucose content was the lowest in CR6 (7.33 mg g^−1^), rose to its highest value of 13.45 in CR7, fell to 7.37 in CR9, and rose to 9.05 mg g^−1^ in CR10. The fruit fructose content was higher than that of sucrose and glucose throughout the whole period with an overall increasing trend from 11.79 in CR6 to 19.08 mg g^−1^ in CR10 (Figure 1E). The leaf sucrose content was below the detection limit in CR6 with content gradually increasing with time (Figure 1D). The highest leaf glucose content of 12.81 mg g^−1^ was observed in CR6 which decreased to 4.12 in CR7 with no significant changes to CR9. The leaf fructose content trend was the same as that of glucose with a maximum of 7.83 in CR6 and a minimum of 2.60 mg g^−1^ in CR7. 

The lowest starch content in the leaves was 19.6 mg g^−1^ in CR6 (Figure 1C) which significantly increased to 30.54 in CR7, significantly decreased in CR9, and increased in CR10 to a similar level as in CR7. Fruit starch content was lower than that of leaves in all periods with an increasing trend from CR6 to CR9 reaching a maximum of 22.55 in CR9 and decreasing to 20.9 mg g^−1^ in CR10 (Figure 1C). 

### 2.3. Sucrose Synthase (SuSy) and Invertase Assays during Fruit Development

**Figure 2:**
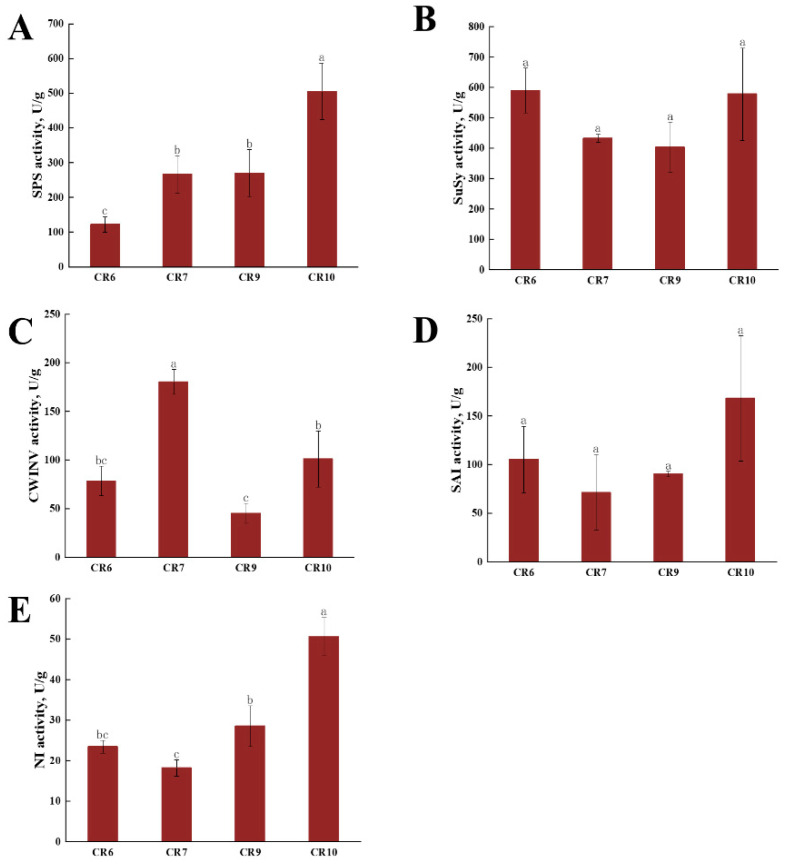
Activities of key enzymes involved in fruit sugar metabolism at different developmental stages. (**A**–**E**) Activity of sucrose phosphate synthase (SPS), sucrose synthase (SuSy), cell wall acid invertase (CWINV), soluble acid invertase (SAI), and neutral invertase (NI), respectively. Significant differences (*p* < 0.05) are indicated by lower case letters. Letters are arranged alphabetically starting from the highest values with similar letters indicating no significant difference. All error bars depict SD of the mean (n ≥ 3).

The lowest SPS activity in CR6 (122.01 μg min g^−1^) rose to 266.22 in CR7, stabilized at 269.9 in CR9, then rapidly increased to 504.76 μg min g^−1^ in CR10 during fruit development (Figure 2A). There was no significant difference in SuSy activity among the four periods, and all remained at a high level. Activity was the highest at the beginning of fruit development, which decreased from CR7 to its lowest activity of 403.84 μg min g^−1^ in CR9 followed by an increase to 578.24 μg min g^−1^ in CR10 (Figure 2B). 

CWINV activity in *C. oleifera* fruit was 78.4 μg min g^−1^ in CR6, reached a peak of 180.42 in CR7, decreased in CR9 to its lowest level of 45.16 μg min g^−1^ then increased again in CR10 (Figure 2C). SAI activity was the lowest in July (CR7), and increased in two periods thereafter, reaching its highest activity of 168.2 μg min g^−1^ in October (CR10) (Figure 2D). NI had the lowest activity compared with the other invertases with a similar trend to soluble acidic invertase and a maximum activity of 50.62 μg min g^−1^ in CR10 (Figure 2E). 

### 2.4. RNA-Seq of C. oleifera Developing Fruit 

**Figure 3:**
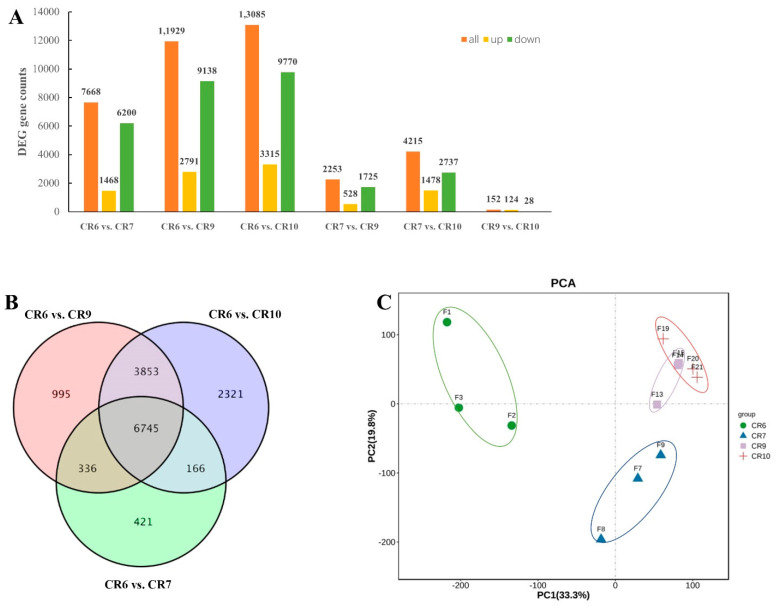
Differentially Expressed Genes (DEGs) of transcriptome sequencing from four fruit developmental stages in *C. oleifera.* (**A**) Summary of DEGS in all combinations of developmental stage comparisons. (**B**) Venn diagram of CR7, CR9, and CR10 compared with CR6, respectively (false discovery rate (FDR) ≤ 0.01, and |log_2_FC| ≥ 1). (**C**) Principal component analysis (PCA) of CR6−CR10. The samples from the same development stage were grouped into the same circle.

**Figure 4:**
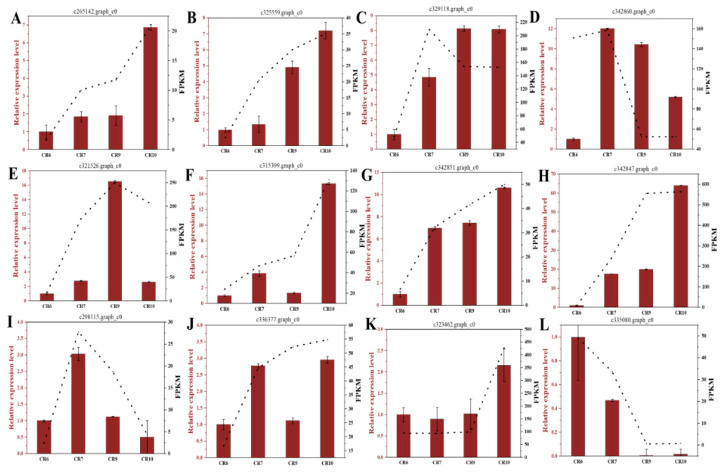
Verification of transcriptomic data by qRT-PCR analysis of 7 candidate gene expression patterns involved in sucrose transport and metabolism from CR6 to CR10 samples. (**A**–**L**) Expression of c265142 (SUC2-like), c325559 (SUT1), c329118 (SUT1), c321526 (SWEET1-like), c315399 (SWEET4), c342851 (SPS), c298115 (SUT3), c336377 (SPS), c323462 (SuSy), and c335080 (SUT1), respectively. The relative expression of target genes compared to a control gene (GAPDH, glyceraldehyde-3-phosphate dehydrogenase) was performed in triplicate with standard errors shown. qPCR values and fragments per kilo base per million mapped reads (FPKM) values from the RNA-seq experiment are shown as bars and lines, respectively.

Transcriptome sequencing of CR6, CR7, CR9, and CR10 fruit development stage generated 90.29 GB of data which was filtered to remove the low-quality reads. Each filtered sample consisted of 5.9 GB high-quality data with a Q30 base percentage of 93.42%. The transcripts of the data were assembled by Trinity software, and 54,217 unigenes were obtained with an average length of 1340.8 bp. This shows that the accuracy and quality of the sequencing data was sufficient for further analysis. The genes in 12 libraries were compared (CR6 vs. CR7, CR6 vs. CR9, CR6 vs. CR10, CR7 vs. CR9, CR7 vs. CR10, and CR9 vs. CR10) to analyze expression differences related to photosynthate distribution and transport in *C. oleifera* seeds in different developmental stages. The differentially expressed genes (DEGs) of CR6 vs. CR10, CR6 vs. CR9, and CR6 vs. CR7 were much larger than those of the other comparisons. Most of the DEGs were down-regulated in the control groups (Figure 3A). The Venn diagram shows that 6745 DEGs overlapped and were stably expressed based on the three comparison groups (Figure 3B). Principal component analysis (PCA) clustered transcripts into four groups, indicating that there were good correlations and differences among the samples (Figure 3C). The consistent gene expression trend of seven DEGs using real-time quantitative reverse transcription polymerase chain reaction (RT-qPCR) analysis (Figure 4) compared with the RNA-seq data verified the transcriptomic results.

### 2.5. Gene Expression Trends during C. oleifera Fruit Development

**Figure 5:**
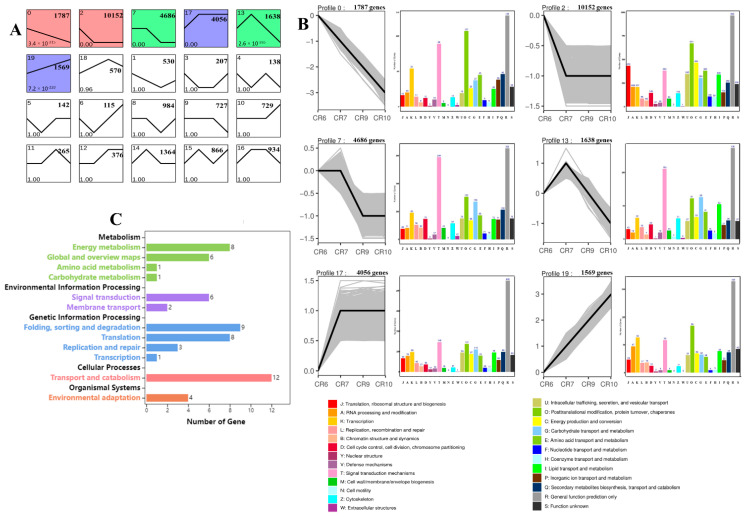
Clusters of total genes and EuKaryotic Orthologous Group (KOG) annotations of profiles. (**A**) All genes were clustered based on their FPKM data with an FDR ≤ 0.05. Profile numbers, *p*-values, and gene numbers are located in the upper left corner, lower left corner, and upper right corner, respectively. (**B**) Six clusters were obtained by short time-series expression miner (STEM) software using the gene expression profiles. The *y*-axis of each cluster indicates the log_2_FC value. A *p* value ≤ 0.05 and a minimum fold change of two were used for KOG analysis. The *y*−axis of each KOG analysis shows the number of genes. (**C**) Kyoto Encyclopedia of Genes and Genomes (KEGG) pathway annotations of profile 13. The *y*−axis of each cluster is the KEGG pathway name.

Twenty gene profiles clustered together based on their expression patterns (Figure 5A). The genes within the top 6 expression profiles were analyzed by KOG (EuKaryotic Orthologous Groups) annotation (Figure 5B). Transcription, translation, ribosomal structure, biogenesis, and signal transduction mechanisms accounted for the majority of differential expression profiles in 2, 7, and 0 indicating that the biological processes related to cell division are slowly declining with growth and development. Post-translational modification, protein turnover, molecular chaperones, carbohydrate transport and metabolism, biosynthesis, transport, and catabolism of secondary metabolites showed an upward trend in profile 17 and 19 indicating that the synthesis and transport of carbohydrate-related sugars and proteins play important roles in fruit ripening. Expression profile 13 showed that signal transduction mechanisms were highly expressed in the early stages (CR6–CR7) of development followed by continuous reduction in CR9 and CR10. Most genes were associated with cellular process such as transport and catabolism by the Kyoto Encyclopedia of Genes and Genomes (KEGG) analysis, which are related to photosynthate transport and metabolism (Figure 5C). The results suggest that the CR7 period may be key during fruit development.

### 2.6. Functional Enrichment Analysis of DEGs

The Gene Ontology (GO) and KEGG databases were used to further analyze DEGs in six comparison groups (CR6 vs. CR7, CR6 vs. CR9, CR6 vs. CR10, CR7 vs. CR9, CR7 vs. CR10, and CR9 vs. CR10) using *p* ≤ 0.01 to indicate a significant difference. The enrichment GO terms (Figure S1) of DEGs to biological processes mainly include metabolic processes, cellular processes and single-organism. The cellular component was mainly enriched in cell, cell part, and membrane. Molecular function was mainly enriched in catalytic activity, binding, transporter activity, and structure molecular activity.

**Figure 6:**
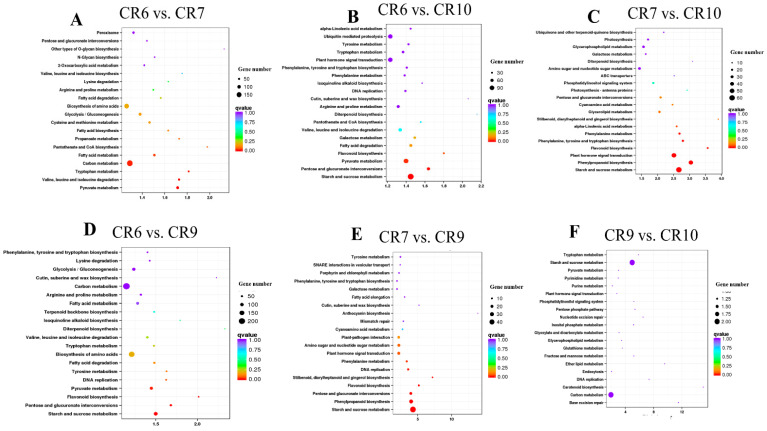
KEGG enrichment of six developmental stage comparisons. (**A**–**F**) Results of KEGG enrichment of differential genes in comparison groups (CR6 vs. CR7, CR6 vs. CR10, CR7 vs. CR10, CR6 vs. CR9, CR7 vs. CR9, and CR9 vs. CR10). The ordinate is the pathway name while the abscissa represents the rich factor. The size of the dot indicates the number of DEGs in the pathway and the color of the dot corresponds to different q-value ranges.

A total of 618 metabolic pathways were involved during the four *C. oleifera* growth and development stages following enriched KEGG analysis of all DEGs in the six comparison groups. DEGs were mainly enriched in carbon metabolism, starch metabolism, and sucrose metabolism (Figure 6, FDR < 0.01).

### 2.7. DEGs Related to Sugar Metabolism

**Figure 7:**
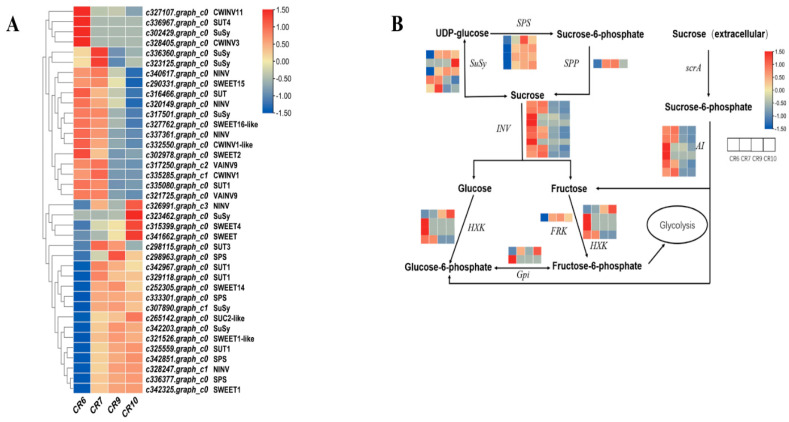
Heat map of DEGs related to sucrose transporters and sucrose metabolic enzymes. (**A**) The heat map was observed by TBtools based on RNA−seq FPKM. The right column shows the corresponding gene IDs and gene names, respectively. The color bar represents the expression level of each gene. (**B**) A simplified sucrose biosynthesis and inversion pathway. Heatmap of columns and rows represents samples and genes, respectively. SPS: sucrose phosphate synthase; SuSy: sucrose synthase; SPP: sucrose phosphatase; INV: invertase; HXK: hexokinase; FRK: fructokinase; Gpi: glucose−6−phosphate isomerase; scrA: sucrose−specific phosphotransferase; AI: acid invertase.

KEGG enrichment of six developmental stage comparisons. The ordinate is the pathway name while the abscissa represents the richness factor. The size of the dot indicates the number of DEGs in the pathway and the color of the dot corresponds to different q-value ranges. DEGs related to sugar metabolism including sucrose metabolism-related enzymes and sugar transport-related proteins were screened from the entire DEG pool (Figure 7A). Cluster heat map analysis divided 38 DEGs into two groups according to their expression levels: class I was highly expressed in the initial period followed by decreased expression while class II had the opposite trend. Class I has six genes encoding sucrose transporter proteins, four encoding sucrose synthase, and nine encoding invertase. All of these genes were highly expressed during CR6 and CR7 with decreased expression in CR9 and CR10. Class II mainly contains genes encoding sucrose transporter proteins and sucrose phosphate synthases. Most of the genes related to sugar metabolism were highly expressed during CR7. There was a significant, positive correlation between the increasing expression of some sugar transporter genes and sucrose content in fruit: low in CR6, highest in CR7, and stable during CR9 and CR10 (Table 1). 

DEGs were significantly enriched in starch and sucrose metabolism in the CR6 vs. CR10 comparison group where most DEGs were found (Figure 6B). c333301.graph_c0 (SPS), c336377.graph_c0 (SPS), c342851.graph_c0 (SPS), c323462.graph_c0 (SuSy), c300533.graph_c0 (HXK; hexokinase), c309252. graph_c0 (Gpi; glucose-6-phosphate isomerase) and other genes were up-regulated (Figure 7B). It is hypothesized that these genes play an important role in sugar synthesis and accumulation during oilseed tea fruit development.

## 3. Discussion

### 3.1. Characteristics of Assimilate Distribution and Accumulation at Different Fruit Developmental Stages

The ^13^C abundance in leaves and fruit was the highest in CR10 and CR7, respectively. The maximum soluble sugar content was in CR7, which implies that rapid expansion of *C. oleifera* fruit may be occurring. The starch content in the fruit increased from CR6 to CR9 (Figure 1C), and reached a maximum in CR9. *C. oleifera* fruit development undergoes oil transformation and ripening during CR9 and CR10, which requires carbon accumulation, so the carbohydrate demand increases. Starch hydrolysis may provide the material basis for oil conversion when the fruit sucrose content is insufficient [33]. Starch hydrolysis creates a temporary total non-structural carbohydrate (TNC) sink caused by sugar demand in fruit exceeding the leaf photo-assimilate supply (TNC source) [34]. The assimilate amount supplied to the sink organ changed during *C. oleifera* development. The order of assimilate supply in CR6, CR7, CR9, and CR10 was branch > leaf > fruit, fruit > branch > leaf, leaf > fruit ≈ branch, and leaf > branch > fruit, respectively. This may be related to the period in which the experiment was conducted: the CR6 period is the young fruit formation stage so the competitiveness of the fruit as a sink may be weak compared with other organs such as roots, which require a large supply of nutrition. Therefore, the carbon nutrients in the leaves may be mainly transported down to the root along the phloem with less movement to the fruit. Its transport is driven by the swelling and pressure difference between the source and the sink ends of the phloem [35,36]. Meanwhile, rapid fruit development during CR7 requires an abundance of nutrients so the competitiveness of fruit as a sink organ in this period is stronger than that of other functional organs. The competitiveness of the sink is lower than that for CR7 in the middle and later stages of fruit development (CR9 and CR10), which involves oil transformation and accumulation. The photosynthates produced by leaves (source organs) are mainly supplied to fruit (sink organs) to meet the physiological and metabolic activities such as volume increase, oil transformation, and seed ripening in development. Recent studies showed that photosynthate accumulation in differently positioned strawberry fruit on the same inflorescence varies in specific leaves and that the photosynthate transport differences between fruit may be due to relative sink activity variances [36]. 

### 3.2. The Relationship between Sucrose Metabolic Enzyme Activity and Soluble Sugar Content 

Fruit soluble sugars mainly include sucrose, glucose, and fructose [37,38]. In the four *C. oleifera* fruit developmental stages, the sucrose content initially increased, then slightly decreased, and finally stabilized, which is consistent with sugar content changes during peach development [14]. Meanwhile, the *C. oleifera* glucose and fructose content showed an overall downward trend. Fructose content was always higher than that of glucose and sucrose and continuously increased in the later developmental stages. There was a positive correlation between fructose content and *C. oleifera* fruit maturity. The presumed constant sucrose synthesis in the leaves as a source organ and the sucrose decomposition in the pulp tissue creates a sucrose concentration difference that ensures the directed transport of photosynthetic products from the source leaves to the sink organ [20,39]. In the early and late stages of *C. oleifera* fruit development, sucrose is transported through the symplast pathway, while in the middle stage, it is transported through the apoplast pathway [35]. CWINV activity peaks in the first pathway, then decreases, and reaches another peak in the second pathway indicating that CWINV plays a role in phloem unloading transformation. The CWINV activity changes during fruit development (Figure 2C) proved that CWINV participates in promoting phloem unloading by the apoplastic pathway. The changes in SAI and CWINV activities were consistent with previous reports showing that SAI plays an active role in vacuolar sucrose metabolism [35]. SAI and NI activities were higher in the later stages of fruit development leading to vacuole expansion and sugar accumulation which promotes *C. oleifera* fruit ripening and oil accumulation [35]. The consistently lower neutral invertase activity compared with the other two invertases during the four growth development stages was inconsistent, which may be due to the particular *C. oleifera* variety since differences in NI activity had been previously observed among different watermelon varieties [40]. Previous studies showed that sucrose content was positively correlated with SuSy (synthetic direction) and SPS activity in other species [41]. In this study, SuSy activity in *C. oleifera* remained at a high level with a slight decrease in activity at CR7, and an increase during CR9 to CR10, but sucrose accumulation did not significantly change during the CR9–CR10 period. We suggest that a large amount of sucrose in *C. oleifera* fruit is converted into glucose and fructose, with glucose used to synthesize oil in the later stages of growth and development [42], while fructose accumulation continuously increased (Figure 1E). In this study, SPS activity showed an upward trend and peaked at CR10. Higher SuSy and SPS activity was observed in the hard-core stage of *C. oleifera* fruits (mid-August) which correlated with the beginning of oil accumulation. Sucrose metabolism, transformation, and oil synthesis were exuberant in this period and the fruit entered a transition period from sugar metabolism to fat and protein metabolism. High sucrose concentration provided the basis for the nutrient transformation and the continually high SuSy and SPS activities during fruit development after CR9 enabled exuberant oil synthesis in this period [43]. There was no conclusive relationship determined between SuSy and SPS activity in *C. oleifera* fruit and sucrose accumulation in this study (Table S1).

### 3.3. Key Genes Related to Sucrose Transport in Fruit

Plant sucrose can be used as a metabolite or as a signal molecule to initiate signaling pathways leading to gene expression changes and physiological adaptation [44]. Expression analysis during *C. oleifera* fruit development and maturation was performed to identify important genes regulating sugar content and transport (Figure 7A). Most of the genes encoding sucrose metabolizing enzymes and sugar transporters were highly expressed during CR7. This correlated with the highest ^13^C abundance at the CR7 stage. Furthermore, ^13^C labeling in fruit was above that of branches and leaves indicating that a large amount of photosynthate was transported from leaves to fruit during this period. In addition, fruit-soluble sugar content was the highest in the CR7 period, implying that this is the key period for sugar synthesis in fruit. Genes are mainly enriched in signal transduction mechanisms according to gene expression profile 13, so it is suggested that sucrose is used as a signaling molecule to initiate related metabolic processes (Figure 5B,C).

Sucrose transporter SUT2 and phosphate transporter Pht1;4 are specifically induced by sucrose in *A. thaliana* [45,46] while BvSUT1 is inhibited by sucrose resulting in decreased sucrose transport in sugar beet [47]. SUTs in plants are divided into three subfamilies (SUT1, SUT2, and SUT3) [2]. Members of the SUT1 subfamily are exclusively located on the plasma membrane and are responsible for loading sucrose into the phloem or absorbing it into sink tissue cells [48,49,50,51]. Most SUT2 subfamily members are located on the plasma membrane of the sugar receptor [52,53]. SUT3 subfamily members are thought to be related to the tonoplast where they participate in sucrose outflow from the vacuole to the cytoplasm [54,55]. Monocotyledons and dicotyledons use different SUT types to load sucrose into the phloem. Dicotyledons rely on the highest sucrose affinity SUT1 subfamily while monocotyledons use high-affinity SUT2 transporters [56,57,58]. *C. oleifera* is a dicotyledonous plant and has four SUTs belonging to the SUT1 subfamily. Gene expression heat maps of *C. oleifera* fruit showed that sucrose transporter expression varied significantly in different developmental stages (Figure 7A). The expression of five SUTs (SUT3, c298115.graph_c0; SUT1, c342967.graph_c0; SUT1, c329118.graph_c0; SUC2-like, c265142.graph_c0; SUT1, c325559.graph_c0) was low at CR6 which correlated with the low sucrose content in this stage. Meanwhile, the sucrose content and the expression of these five SUTs increased in CR7. The expression of four SUTs (except SUT3, c298115.graph_c0) decreased when the sucrose content in the fruit reached its peak and were maintained at stable levels which played an important role in the dynamic balance of sucrose. The four SUTs were significantly positively correlated with sucrose content (Table 1) which suggests that they may be the key genes responsible for sucrose transport in fruit. 

SWEET family members are sugar efflux transporters and the number of SWEET protein members is important for plant physiological development, including phloem loading and unloading and other processes [10,59,60]. AtSWEET15 is involved in sugar transport from the phloem to the sink organs in *A. thaliana* and specifically involving the release of sucrose from the seed coat to the cytoplasm to supply seed maturation [61]. AtSWEET16 is located on the tonoplast and plays an important role in maintaining the dynamic sugar balance, but its function is unclear [10,62]. SWEET-like transporters are bidirectional, which means they monitor the inflow of sugar and control its outflow [63]. Five SWEET genes were up-regulated to promote sugar flux between intercellular space or between mesophyll cells and phloem during *C. oleifera* fruit development; however, *SWEET15*, *SWEET2* and *SWEET16*-like gene expression was down-regulated (Figure 7A) indicating that these transporters may be used as exporters to pump sugar and reduce its concentration from the plasma membrane and tonoplast. 

### 3.4. Relationship between Sugar Accumulation and Sucrose Metabolism Enzyme Transcripts 

Sucrose is involved in complex metabolic processes during *C. oleifera* fruit development. The DEGs in the CR6vsCR10 comparison group were significantly enriched in starch and sucrose metabolism (Figure 6B) and 26 genes related to sucrose metabolism were identified encoding INV, Susy, SPS, SPP (sucrose phosphatase), HXK, FRK (fructokinase), and others according to the KEGG enrichment pathway (Figure 7A). Sucrose content is regulated by positive (synthesis) and negative (decomposition) of genes related to sucrose metabolism [44,64]. The sharp increase in sucrose content is accompanied by a sharp increase in *SPS* expression during pineapple fruit [65] and apple development [23]. In addition, high SuSy expression promotes sucrose accumulation in *Pyrus* [66]. Four DEGs encoding SPS (c298963.graph_c0, c333301.graph_c0, c336377.graph_c0, c342851.graph_c0) and three genes encoding SuSy (c307890.graph_c1, c323462.graph_c0, c342203.graph_c0) were highly expressed during *C. oleifera* fruit development which contributed to the final sucrose content (Figure 7B). Both SuSy and INV decompose sucrose: SuSy reversibly hydrolyzes sucrose into UDPG (uridine-5’-diphosphate-glucose) and fructose [8]; INV (CWIN, NI, and SAI) irreversibly hydrolyzes sucrose producing glucose and fructose [21]. The two SuSy subtypes in Japanese pear fruit (SuSy I and SuSy II) are involved in sucrose degradation and synthesis, respectively [67]. The expression of 6 genes encoding AI (c317250.graph_c2, c321725.graph_c0, c327107.graph_c0, c328405.graph_c0, c332550.graph_c0, c335285.graph_c1) and two genes encoding SuSy c317501.graph_c0, c336360.graph_c0) were down-regulated in the late developmental stage, suggesting that the two SuSy proteins SAI and CWINV may function in sucrose decomposition. This shows that low levels of sucrose degradation occurs despite the overall increase in sucrose observed during development. The decrease in *OsVIN2* expression significantly increases sucrose content in rice seeds [68]. Three genes encoding NI (c337361.graph_c0, c328247.graph_c1, c340617.graph_c0) had relatively high expression during the four *C. oleifera* fruit development periods although they were not enriched in the pathways related to sucrose metabolism, and NI activity was significantly lower than that of the other two invertase enzymes. The role of NI in *C. oleifera* fruit cannot be deduced from this study. SuSy (synthesis) and SPS are mainly responsible for the synthesis and accumulation of sucrose and the expression increases in the later stage of *C. oleifera* fruit development although invertase is also involved. This correlated with previous studies in *Brassica napus* L. showing that invertase produced both glucose and fructose in the fruit, while SuSy produced fructose in the late development stage [42]. *FRK* expression decreased in the later stages of fruit development indicating that the fructose content increased during this stage, which is consistent with decreased *FRK* expression and fructose content observed in apples [23].

**Figure 8:**
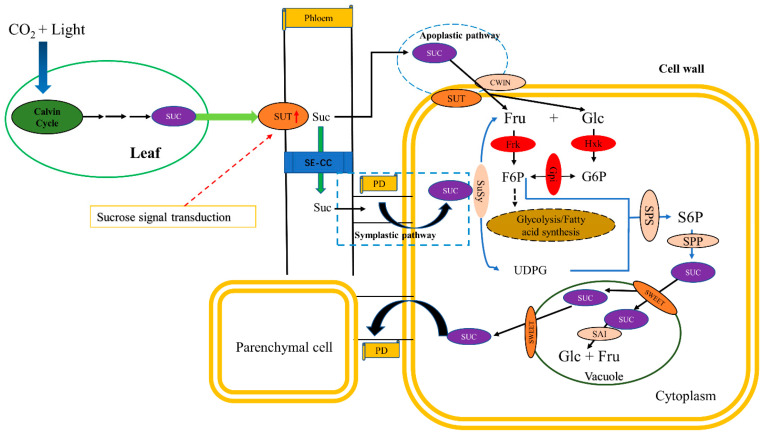
Hypothetical model of sucrose synthesis, transport, and metabolism in *C. oleifera* fruit development. Sucrose is synthesized in leaves and initiates signal transduction which induces SUT expression. Sucrose molecules are loaded in the phloem and enter sink cells by two different transport pathways: symplastic and apoplastic. Changes in sucrose content are controlled under the combined action of sucrose synthesis and invertase in different developmental stages. SUC: sucrose; SUT: sucrose transporter; SE/CC: sieve element/companion cell; PD: plasmodesmata; CWIN: cell wall acid invertase; Fru: fructose; Glc: glucose; F6P: fructose−6−phosphate; G6P: glucose−6−phosphate; Frk: fructokinase; Hxk: hexokinase; Gpi: glucose−6−phosphate isomerase; SuSy: sucrose synthase; SPS: sucrose phosphate synthase; S6P: sucrose−6−phosphate; SPP: sucrose phosphatase; SWEET: sugars will eventually be exported transporters; SAI: soluble acid invertase; UDPG: uridine−5′−diphosphate−glucose.

A model of sucrose transport and metabolism in *C. oleifera* fruit is presented based on this study and previous literature (Figure 8). There are two sucrose transport pathways in *C. oleifera* fruit: the symplast and the apoplast. The symplast pathway is dependent on plasmodesmata (PD) while the apoplast pathway is dependent on SUT, and the intracellular sucrose content is mainly regulated by SPS and AI. The comprehensive transcriptomic, enzymatic, and sugar analysis information provided in this study serves as a powerful resource for understanding sugar metabolism, sucrose synthesis, transport and invertase related genes and enzymes during *C. oleifera* fruit ripening.

## 4. Materials and Methods

### 4.1. Plant Materials

*C. oleifera* Abel. fruit (variety ‘CenRuan 3’) was collected from Xiaokeng Forest Farm, Qujiang District, Shaoguan City, Guangdong Province, China (113°35′08″ E, 24°15′42″ N), with an average tree height of 2.43 ± 0.03 m and an average ground diameter of 85.41 ± 0.63 mm. The average weight, longitudinal diameter, and transverse diameter of the fruit was 36.08 ± 0.99 g, 38.32 ± 2.59 mm, and 42.47 ± 2.38 mm, respectively. Fifteen trees were selected for ^13^C labeling and soluble sugar content determination and three trees were selected for transcriptome sequencing. The fruit samples were collected in June (CR6), July (CR7), September (CR9), and October (CR10) in 2019, wrapped in tin foil, and placed into liquid nitrogen before storage (−80 °C) and laboratory analysis.

### 4.2. ^13^C Pulse-Labeling and Carbon Abundance Determination

Three sample trees (representing three replicates) were selected from 15 trees in sunny weather. One branch of each sample tree retaining the same number of leaves per branch and fruit number and size was selected for the experiment. The middle leaves were selected, bagged, and fed with ^13^C using a previous experimental method with slight modifications [69]. The polyethylene plastic bag had good light transmittance. The mouth of the bag was fastened and sealed with professional sealing mud, then the air was removed by a vacuum pump (Dryfasteco, Welch, Co., Chicago, IL, USA) and injected with air lacking CO_2_ and H_2_O, which was absorbed by sodium hydroxide, and injected with ^13^CO_2_ gas (99 atom% ^13^C) with a 10 mL syringe to keep the ^13^CO_2_ concentration in the bag at 500 μmol·mol^−1^. The feeding was stopped after 1 h and the bag was removed. Leaves, branches, and fruit were collected and placed in envelopes 72 h after labeling. At the same time, three other sample trees representing the control samples were selected without ^13^CO_2_ feeding and sampled in the same manner.

The leaf, branch, and fruit samples were placed in an oven for inactivation at 105 °C for 30 min, then dried at 60 °C until the weight was consistent. Samples were crushed with a mortar and pestle and passed through a 100-mesh sieve. ^13^C abundance and total carbon content was determined by Shenzhen Huake Jingxin Isotope Laboratory.

### 4.3. Soluble Sugar Content Measurement 

Healthy leaves and fruit, dried as described in the previous section from four growth periods, were ground into powdered samples using a mortar and pestle. Fifty milligrams of dried sample was placed into a 10 mL centrifuge tube, then 4 mL of 80% aqueous ethanol solution was added and the mixture was incubated in an 80 °C water bath for 30 min. The sample was centrifuged at 8500 rpm for 5 min at 25 °C, and the supernatant was transferred to a 50 mL volumetric flask. Then 4 mL of 80% aqueous ethanol solution was added to the substrate precipitation and extracted again. The extraction was repeated three times with the supernatants merged into the 50 mL volumetric flask, and the volume adjusted to 50 mL with 80% aqueous solution [70]. The total soluble sugar content was determined using the anthrone–sulfuric acid colorimetric method [71] measured at 625 nm using a MAPADA^®^ UV-1200 spectrophotometer (Shanghai Mapada Instruments Co., Ltd., Shanghai, China). 

### 4.4. Determination of Starch Content

The fruit and leaves of *C. oleifera* stored at −80 °C were ground in liquid nitrogen, and a 0.1 g sample was taken to determine the starch content (a 1:10 ratio of tissue mass to extraction volume) using a starch content test kit following the manufacturer’s instructions (Suzhou Keming Biotechnology Co., Ltd., Suzhou, China). The sample was first mixed with 1 mL of 80% ethanol aqueous solution and the mixture was incubated in an 80 °C water bath for 30 min. The sample was centrifuged at 3000 rpm for 5 min at 25 °C. The supernatant was discarded, 0.5 mL of distilled water was added to the precipitate, and the sample was bathed in water at 95 °C for 15 min. After cooling, 0.35 mL of 9.2 M HClO_4_ was added, and the sample was extracted at 25 °C for 15 min. Then 0.85 mL of distilled water was added to the mixture, mixed well and centrifuged at 3000 rpm for 10 min at 25 °C. Fifty microliters of the supernatant and 250 μL of 2% anthraquinone reagent were taken into a centrifuge tube, mixed and then bathed in 95 °C water for 10 min. After cooling, the 200 μL solution was used to measure the starch content at 620 nm using a Multiskan FC microplate reader (Thermo Fisher Scientific, Waltham, MA, USA). Five replicates were used for each experiment.

### 4.5. Determination of Sugar Content

Frozen samples were ground with liquid nitrogen and 1 g was used for the assay. Double-distilled water (5 mL) was added and the mixture was heated in an 80 °C water bath for 20 min. The sample was cooled for 15 min, then centrifuged at 3000 rpm for 15 min at 25 °C. The supernatant was collected and the extraction of the pellet was repeated once using 4 mL of double-distilled water. The supernatants were combined and the volume adjusted to 10 mL. The content of sucrose, glucose and fructose was determined by high-performance liquid chromatography (HPLC) according to a previous method [72]. The sample injection volume and flow rate of the mobile phase (ultrapure water) was 10 μL and 0.3 mL·min^−1^, respectively. The Agilent Hi-Plex Ca column had an inner diameter of 4.0 × 300 mm and a particle size of 8 μm. The column temperature was maintained at 80 °C. All sugar content were determined according to an external standard solution purchased from Sigma Chemical Co., St. Louis, MO, USA. The concentration of each sample was calculated by comparing the peak area and retention time with those of known calibrated sugar solutions concentrations.

### 4.6. Sucrose Metabolism Enzyme Activity Assays

*C. oleifera* fruit samples stored at −80 °C were ground in liquid nitrogen, and 0.1 g of sample was weighed for enzyme activity determination at a tissue mass to extraction solution volume ratio of 1:5–10. The enzyme activities of SuSy, SPS, NI, CWINV, and SAI were measured by SS assay kit (G0512W), SPS assay kit (G0515W), NI assay kit (G0516W), CWINV assay kit (G0518W), and SAI assay kit (G0517W) purchased from Suzhou Grace Biotechnology Co. Ltd. (Suzhou, China). Each experiment was repeated five times.

### 4.7. RNA Extraction and RNA-seq Library Construction and Sequencing

Transcriptome RNA library construction and RNA sequencing were carried out using triplicate samples of CR6, CR7, CR9 and CR10 using a plant RNA isolation kit (Omega Bio-Tek Co., Norcross, GA, USA). RNA purity, concentration, and integrity was tested using NanoPhotometer N60 (Implen, Munich, Germany)to ensure the use of qualified samples for transcriptome sequencing. Library construction and RNA-seq was performed by Biomarker Technologies Co., Ltd. (Beijing, China) using one μg of RNA per sample. Sequencing libraries were generated using the NEBNext^®^Ultra™ RNA Library Prep Kit for Illumina^®^ (NEB, Ipswich, MA, USA) following the manufacturer’s recommendations and index codes were added to attribute sequences to each sample. Briefly, mRNA was purified from total RNA using poly-T oligo-attached magnetic beads. Fragmentation was carried out using divalent cations under elevated temperature in NEBNext First Strand Synthesis reaction buffer (5X). First strand cDNA was synthesized using random hexamer primers and M-MuLV Reverse Transcriptase (Thermo Fisher Scientific, Waltham, MA, USA). Second-strand cDNA synthesis was subsequently performed using DNA Polymerase I and RNase H. The remaining overhangs were converted into blunt ends via exonuclease/polymerase activities. After adenylation of 3′ ends of DNA fragments, NEBNext Adaptor with hairpin loop structure was ligated to prepare for hybridization. The library fragments were purified with the AMPure XP system (Beckman Coulter, Beverly, MA, USA) to select cDNA fragments 240 bp long. Three μL of USER enzyme (NEB, USA) was added with size-selected, adapter-ligated cDNA at 37 °C for 15 min followed by 5 min at 95 °C. A PCR test was then performed using Phusion High-Fidelity DNA polymerase (Thermo Fisher Scientific, Waltham, MA, USA), universal PCR primers, and Index (X) Primer. PCR products were purified (AMPure XP system) and the library quality was assessed by an Agilent 2100 Bioanalyzer system. Clustering of the index-coded samples was performed on an Illumina cBot cluster generation system using the TruSeq PE cluster kit v.4-cBot-HS (Illumina) according to the manufacturer’s instructions. The libraries were sequenced on an Illumina platform and paired-end reads were generated. The adaptor sequences and low-quality sequence reads were removed from the datasets after quality control. Raw sequences were transformed into clean reads after data processing. Gene function was annotated from databases including NR (NCBI non-redundant protein sequences); NT (NCBI nonredundant nucleotide sequences); Pfam (Protein family); KOG/COG (Clusters of Orthologous Groups of proteins); Swiss-Prot (a manually annotated and reviewed protein sequence database); KO (KEGG Orthologue database); and GO (Gene Ontology) resources. DEGs were observed using BMKCloud with DESeq2 and EBSeq software and parameters included a false discovery rate (FDR) ≤ 0.05 and log_2_FCa (FC, fold change) ≥ 1.

### 4.8. Quantitative RT-PCR Analysis

The TUREscript 1st Stand cDNA synthesis kit reverse transcription kit (Aidlab Biotechnologies Co., Ltd., Beijing, China) was used to synthesize cDNA for qRT-PCR analysis on an qTOWER 2.2 fluorescent quantitative PCR instrument (Analytik Jena, Jena, Germany). The reaction procedure was 95 °C, 30 s; followed by 40 cycles of 95 °C, 5 s; 5 °C, 30 s; 72 °C, 20 s. The relative expression of each gene was calculated by the 2^−∆∆Ct^ method [73] using *GAPDH* (*glyceraldehyde-3-phosphate dehydrogenase*) as an internal reference gene, and three replicates of each reaction were performed [74]. The gene-specific primers are listed in Table S2.

## Figures and Tables

**Table 1:** Correlation analysis between gene expression and sucrose content.

	c342967.graph_c0 (SUT1)	c329118.graph_c0 (SUT1)	c265142.graph_c0 (SUC2-like)	c321526.graph_c0 (SWEET1-like)	c325559.graph_c0 (SUT1)
Sucrose	0.752 **	0.756 **	0.599 *	0.738 **	0.675 *

* and ** represents *p* < 0.05 and *p* < 0.01, respectively.

## Data Availability

RNA-seq data were submitted to https://bigd.big.ac.cn/gsub/ (accessed on 14 December 2021) and the submission number was CRA005580.

## Supplementary Materials

The following are available online at https://www.mdpi.com/article/10.3390/ijms23020822/s1.

## References

[1] Davis J.P., Dean L.L., Stalker H.T., Wilson R.F. (2016). Chapter 11—Peanut composition, flavor and nutrition. Peanuts.

[2] Kühn C., Grof C.P. (2010). Sucrose transporters of higher plants. Curr. Opin. Plant Biol..

[3] Wiley E., Huepenbecker S., Casper B.B., Helliker B.R. (2013). The effects of defoliation on carbon allocation: Can carbon limitation reduce growth in favour of storage?. Tree Physiol..

[4] Yue C., Cao H.L., Wang L., Zhou Y.H., Huang Y.T., Hao X.Y., Wang Y.C., Wang B., Yang Y.J., Wang X.C. (2015). Effects of cold acclimation on sugar metabolism and sugar-related gene expression in tea plant during the winter season. Plant Mol. Biol..

[5] Stitt M. (2013). Progress in understanding and engineering primary plant metabolism. Curr. Opin. Biotechnol..

[6] Rosche E., Blackmore D., Tegeder M., Richardson T., Schroeder H., Higgins T.J., Frommer W.B., Offler C.E., Patrick J.W. (2010). Seed-specific overexpression of a potato sucrose transporter increases sucrose uptake and growth rates of developing pea cotyledons. Plant J..

[7] Ainsworth E.A., Bush D.R. (2011). Carbohydrate export from the leaf: A highly regulated process and target to enhance photosynthesis and productivity. Plant Physiol..

[8] Ren R., Yue X., Li J., Xie S., Guo S., Zhang Z. (2020). Coexpression of sucrose synthase and the SWEET transporter, which are associated with sugar hydrolysis and transport, respectively, increases the hexose content in *Vitis vinifera* L. grape berries. Front. Plant Sci..

[9] Chen L.Q., Hou B.H., Lalonde S., Takanaga H., Hartung M.L., Qu X.Q., Guo W.J., Kim J.G., Underwood W., Chaudhuri B. (2010). Sugar transporters for intercellular exchange and nutrition of pathogens. Nature.

[10] Chardon F., Bedu M., Calenge F., Klemens P.A.W., Spinner L., Clement G., Chietera G., Léran S., Ferrand M., Lacombe B. (2013). Leaf fructose content is controlled by the vacuolar transporter SWEET17 in *Arabidopsis*. Curr. Biol..

[11] Peng Q., Cai Y., Lai E., Nakamura M., Liao L., Zheng B., Ogutu C., Cherono S., Han Y. (2020). The sucrose transporter MdSUT4.1 participates in the regulation of fruit sugar accumulation in apple. BMC Plant Biol..

[12] Li F., Yan L., Lai J., Ma C., Gautam M., Fu T. (2013). Molecular cloning and mRNA expression profile of sucrose transporter gene *BnSUT1C* from *Brassica napus* L. Indian J. Exp. Biol..

[13] Aguirre M., Kiegle E., Leo G., Ezquer I. (2018). Carbohydrate reserves and seed development: An overview. Plant Reprod..

[14] Aslam M.M., Deng L., Wang X., Wang Y., Pan L., Liu H., Niu L., Lu Z., Cui G., Zeng W. (2019). Expression patterns of genes involved in sugar metabolism and accumulation during peach fruit development and ripening. Sci. Hortic..

[15] Garcia-Gomez B.E., Ruiz D., Salazar J.A., Rubio M., Martinez-Garcia P.J., Martinez-Gómez P. (2020). Analysis of metabolites and gene expression changes relative to apricot (*Prunus armeniaca* L.) fruit quality during development and ripening. Front. Plant Sci..

[16] Zhang Q., Feng C., Li W., Qu Z., Zeng M., Xi W. (2019). Transcriptional regulatory networks controlling taste and aroma quality of apricot (*Prunus armeniaca* L.) fruit during ripening. BMC Genom..

[17] Ma Q.-J., Sun M.-H., Liu Y.-J., Lu J., Hu D.-G., Hao Y.-J. (2016). Molecular cloning and functional characterization of the apple sucrose transporter gene *MdSUT2*. Plant Physiol. Biochem..

[18] Wang J.-G., Zhao T.-T., Wang W.-Z., Feng C.-L., Feng X.-Y., Xiong G.-R., Shen L.-B., Zhang S.-Z., Wang W.-Q., Zhang Z.-X. (2019). Culm transcriptome sequencing of Badila (*Saccharum officinarum* L.) and analysis of major genes involved in sucrose accumulation. Plant Physiol. Biochem..

[19] Zhang C., Zhang H., Zhan Z., Liu B., Chen Z., Liang Y. (2016). Transcriptome analysis of sucrose metabolism during bulb swelling and development in onion (*Allium cepa* L.). Front. Plant Sci..

[20] Fugate K.K., Eide J.D., Martins D.N., Grusak M.A., Deckard E.L., Finger F.L. (2019). Colocalization of sucrose synthase expression and sucrose storage in the sugarbeet taproot indicates a potential role for sucrose catabolism in sucrose accumulation. J. Plant Physiol..

[21] Li W., Huang L., Liu N., Pandey M.K., Chen Y., Cheng L., Guo J., Yu B., Luo H., Zhou X. (2021). Key regulators of sucrose metabolism identified through comprehensive comparative transcriptome analysis in peanuts. Int. J. Mol. Sci..

[22] Yang Z., Wang T., Wang H., Huang X., Qin Y., Hu G. (2013). Patterns of enzyme activities and gene expressions in sucrose metabolism in relation to sugar accumulation and composition in the aril of *Litchi chinensis* Sonn. J. Plant Physiol..

[23] Li M., Feng F., Cheng L. (2012). Expression patterns of genes involved in sugar metabolism and accumulation during apple fruit development. PLoS ONE.

[24] Zhuang R. (2008). Comprehensive Utilization of Tea-Oil Fruits. Tea-Oil Tree (Camellia oleifera Abel) of China.

[25] Feng J.-L., Yang Z.-J., Chen S.-P., El-Kassaby Y.A., Chen H. (2017). High throughput sequencing of small RNAs reveals dynamic microRNAs expression of lipid metabolism during *Camellia oleifera* and *C. meiocarpa* seed natural drying. BMC Genom..

[26] He L., Guo-ying Z., Huai-yun Z., Jun-ang L. (2011). Research progress on the health function of tea oil. J. Med. Plant Res..

[27] Cheng Y.-T., Lu C.-C., Yen G.-C. (2015). Beneficial effects of camellia oil (*Camellia oleifera* Abel.) on hepatoprotective and gastroprotective activities. J. Nutr. Sci. Vitaminol..

[28] Domisch T., Finér L., Lehto T. (2001). Effects of soil temperature on biomass and carbohydrate allocation in Scots pine (*Pinus sylvestris*) seedlings at the beginning of the growing season. Tree Physiol..

[29] Ngugi M.R., Hunt M.A., Doley D., Ryan P., Dart P. (2003). Dry matter production and allocation in *Eucalyptus cloeziana* and *Eucalyptus argophloia* seedlings in response to soil water deficits. New For..

[30] Zang U., Goisser M., Grams T.E.E., Häberle K.-H., Matyssek R., Matzner E., Borken W. (2014). Fate of recently fixed carbon in European beech (*Fagus sylvatica*) saplings during drought and subsequent recovery. Tree Physiol..

[31] Sun Z.-A., Chen Q., Han X., Wu W.-L., Meng F.-Q. (2018). Estimation of winter wheat photosynthesized carbon distribution and allocation belowground via ^13^C pulse-labeling. Huan Jing Ke Xue.

[32] Troncoso-Ponce M.A., Garcés R., Martínez-Force E. (2010). Glycolytic enzymatic activities in developing seeds involved in the differences between standard and low oil content sunflowers (*Helianthus annuus* L.). Plant Physiol. Biochem..

[33] Hajduch M., Matusova R., Houston N.L., Thelen J.J. (2011). Comparative proteomics of seed maturation in oilseeds reveals differences in intermediary metabolism. Proteomics.

[34] Eveland A.L., Jackson D.P. (2012). Sugars, signalling, and plant development. J. Exp. Bot..

[35] Wang X., You H., Yuan Y., Zhang H., Zhang L. (2018). The cellular pathway and enzymatic activity for phloem-unloading transition in developing *Camellia oleifera* Abel. fruit. Acta Physiol. Plant..

[36] Hidaka K., Miyoshi Y., Ishii S., Suzui N., Yin Y.-G., Kurita K., Nagao K., Araki T., Yasutake D., Kitano M. (2018). Dynamic analysis of photosynthate translocation into strawberry fruits using non-invasive ^11^C-labeling supported with conventional destructive measurements using ^13^C-Labeling. Front. Plant Sci..

[37] Rai M.K., Shekhawat N.S. (2014). Recent advances in genetic engineering for improvement of fruit crops. Plant Cell, Tiss. Organ Cult..

[38] Iqbal S., Ni X., Bilal M.S., Shi T., Khalil-Ur-Rehman M., Zhenpeng P., Jie G., Usman M., Gao Z. (2020). Identification and expression profiling of sugar transporter genes during sugar accumulation at different stages of fruit development in apricot. Gene.

[39] Zhu X., Wang M., Li X., Jiu S., Wang C., Fang J. (2017). Genome-wide analysis of the sucrose synthase gene family in grape (*Vitis vinifera*): Structure, evolution, and expression profiles. Genes.

[40] Liu J., Guo S., He H., Zhang H., Gong G., Ren Y., Xu Y. (2013). Dynamic characteristics of sugar accumulation and related enzyme activities in sweet and non-sweet watermelon fruits. Acta Physiol. Plant..

[41] Burger Y., Schaffer A.A. (2007). The contribution of sucrose metabolism enzymes to sucrose accumulation in *Cucumis melo*. J. Am. Soc. Hortic. Sci..

[42] Hill L.M., Morley-Smith E.R., Rawsthorne S. (2003). Metabolism of sugars in the endosperm of developing seeds of oilseed rape. Plant Physiol..

[43] Zhang L.-Y., Wang X.-Y., Cao Y.-B. (2013). Soluble sugar content and key enzyme activity and the relationship between sugar metabolism and lipid accumulation in developing fruit of *Camellia oleifera*. J. Beijing For. Univ..

[44] Wind J., Smeekens S., Hanson J. (2010). Sucrose: Metabolite and signaling molecule. Phytochemistry.

[45] Barker L., Kühn C., Weise A., Schulz A., Gebhardt C., Hirner B., Hellmann H., Schulze W., Ward J.M., Frommer W.B. (2000). SUT2, a putative sucrose sensor in sieve elements. Plant Cell.

[46] Karthikeyan A.S., Varadarajan D.K., Jain A., Held M.A., Carpita N.C., Raghothama K.G. (2007). Phosphate starvation responses are mediated by sugar signaling in *Arabidopsis*. Planta.

[47] Vaughn M.W., Harrington G.N., Bush D.R. (2002). Sucrose-mediated transcriptional regulation of sucrose symporter activity in the phloem. Proc. Natl. Acad. Sci. USA.

[48] Öner-Sieben S., Lohaus G. (2014). Apoplastic and symplastic phloem loading in *Quercus robur* and *Fraxinus excelsior*. J. Exp. Bot..

[49] Öner-Sieben S., Rappl C., Sauer N., Stadler R., Lohaus G. (2015). Characterization, localization, and seasonal changes of the sucrose transporter FeSUT1 in the phloem of *Fraxinus excelsior*. J. Exp. Bot..

[50] Nieberl P., Ehrl C., Pommerrenig B., Graus D., Marten I., Jung B., Ludewig F., Koch W., Harms K., Flügge U.-I. (2017). Functional characterisation and cell specificity of BvSUT 1, the transporter that loads sucrose into the phloem of sugar beet (*Beta vulgaris* L.) source leaves. Plant Biol..

[51] Dobbelstein E., Fink D., Öner-Sieben S., Czempik L., Lohaus G. (2019). Seasonal changes of sucrose transporter expression and sugar partitioning in common European tree species. Tree Physiol..

[52] Ma Q.-J., Sun M.-H., Lu J., Liu Y.-J., Hu D.-G., Hao Y.-J. (2017). Transcription factor AREB2 is involved in soluble sugar accumulation by activating sugar transporter and amylase genes. Plant Physiol..

[53] Ma Q.-J., Sun M.-H., Lu J., Kang H., You C.-X., Hao Y.-J. (2019). An apple sucrose transporter MdSUT2. 2 is a phosphorylation target for protein kinase MdCIPK22 in response to drought. Plant Biotechnol..

[54] Schneider S., Hulpke S., Schulz A., Yaron I., Höll J., Imlau A., Schmitt B., Batz S., Wolf S., Hedrich R. (2012). Vacuoles release sucrose *via* tonoplast-localised SUC4-type transporters. Plant Biol..

[55] Zanon L., Falchi R., Hackel A., Kühn C., Vizzotto G. (2015). Expression of peach sucrose transporters in heterologous systems points out their different physiological role. Plant Sci..

[56] Gottwald J.R., Krysan P.J., Young J.C., Evert R.F., Sussman M.R. (2000). Genetic evidence for the in planta role of phloem-specific plasma membrane sucrose transporters. Proc. Natl. Acad. Sci. USA.

[57] Sun Y., Reinders A., LaFleur K.R., Mori T., Ward J.M. (2010). Transport activity of rice sucrose transporters OsSUT1 and OsSUT5. Plant Cell Physiol..

[58] Slewinski T.L., Meeley R., Braun D.M. (2009). Sucrose transporter1 functions in phloem loading in maize leaves. J. Exp. Bot..

[59] Chen L.-Q., Qu X.-Q., Hou B.-H., Sosso D., Osorio S., Fernie A.R., Frommer W.B. (2012). Sucrose efflux mediated by SWEET proteins as a key step for phloem transport. Science.

[60] Li J., Chen D., Jiang G.-L., Song H.-Y., Tu M.-Y., Sun S.-X. (2020). Molecular cloning and expression analysis of *EjSWEET15*, enconding for a sugar transporter from loquat. Sci. Hortic..

[61] Chen L.-Q., Lin I.W., Qu X.-Q., Sosso D., McFarlane H.E., Londoño A., Samuels A.L., Frommer W.B. (2015). A cascade of sequentially expressed sucrose transporters in the seed coat and endosperm provides nutrition for the *Arabidopsis* embryo. Plant Cell.

[62] Guo W.-J., Nagy R., Chen H.-Y., Pfrunder S., Yu Y.-C., Santelia D., Frommer W.B., Martinoia E. (2014). SWEET17, a facilitative transporter, mediates fructose transport across the tonoplast of *Arabidopsis* roots and leaves. Plant Physiol..

[63] Chen L.-Q., Cheung L.S., Feng L., Tanner W., Frommer W.B. (2015). Transport of sugars. Annu. Rev. Biochem..

[64] Stein O., Granot D. (2019). An overview of sucrose synthases in plants. Front. Plant Sci..

[65] Zhang X.-M., Wang W., Du L.-Q., Xie J.-H., Yao Y.-L., Sun G.-M. (2012). Expression patterns, activities and carbohydrate-metabolizing regulation of sucrose phosphate synthase, sucrose synthase and neutral invertase in pineapple fruit during development and ripening. Int. J. Mol. Sci..

[66] Lü J., Tao X., Yao G., Zhang S., Zhang H. (2020). Transcriptome analysis of low-and high-sucrose pear cultivars identifies key regulators of sucrose biosynthesis in fruits. Plant Cell Physiol..

[67] Tanase K., Yamaki S. (2000). Sucrose synthase isozymes related to sucrose accumulation during fruit development of Japanese pear (*Pyrus pyrifolia* Nakai). J. Jpn. Soc. Hortic. Sci..

[68] Lee D.-W., Lee S.-K., Rahman M.M., Kim Y.-J., Zhang D., Jeon J.-S. (2019). The role of rice vacuolar invertase2 in seed size control. Mol. Cells.

[69] An T., Schaeffer S., Li S., Fu S., Pei J., Li H., Zhuang J., Radosevich M., Wang J. (2015). Carbon fluxes from plants to soil and dynamics of microbial immobilization under plastic film mulching and fertilizer application using ^13^C pulse-labeling. Soil Biol. Biochem..

[70] Wei J., Qi X., Zhu X., Ma F. (2009). Relationship between the characteristics of sugar accumulation and fruit quality in apple (*Malus domestica* Borkh.) fruit. Acta Bot. Sin..

[71] Yemm E.W., Willis A.J. (1954). The estimation of carbohydrates in plant extracts by anthrone. Biochem. J..

[72] Sturm K., Koron D., Stampar F. (2003). The composition of fruit of different strawberry varieties depending on maturity stage. Food Chem..

[73] Pfaffl M.W. (2001). A new mathematical model for relative quantification in real-time RT–PCR. Nucleic Acids Res..

[74] Zhang W., Ruan C., Li J. (2018). Screening of reference genes in four woody-oil trees and spatio-temporal expression analysis of actin gene. Mol. Plant Breed..

