# Supplementary material for: Sugar Metabolism and Transcriptome Analysis Reveal Key Sugar Transporters during Camellia oleifera Fruit Development
Source: Int J Mol Sci. 2022 Jan 13;23(2):822. doi: 10.3390/ijms23020822 (PMC8775869; doi:10.3390/ijms23020822)
Supplement: Supplementary file 1 [file ijms-23-00822-s001.zip › Figure S1 GO enrichment of six developmental stage comparisons.pdf]

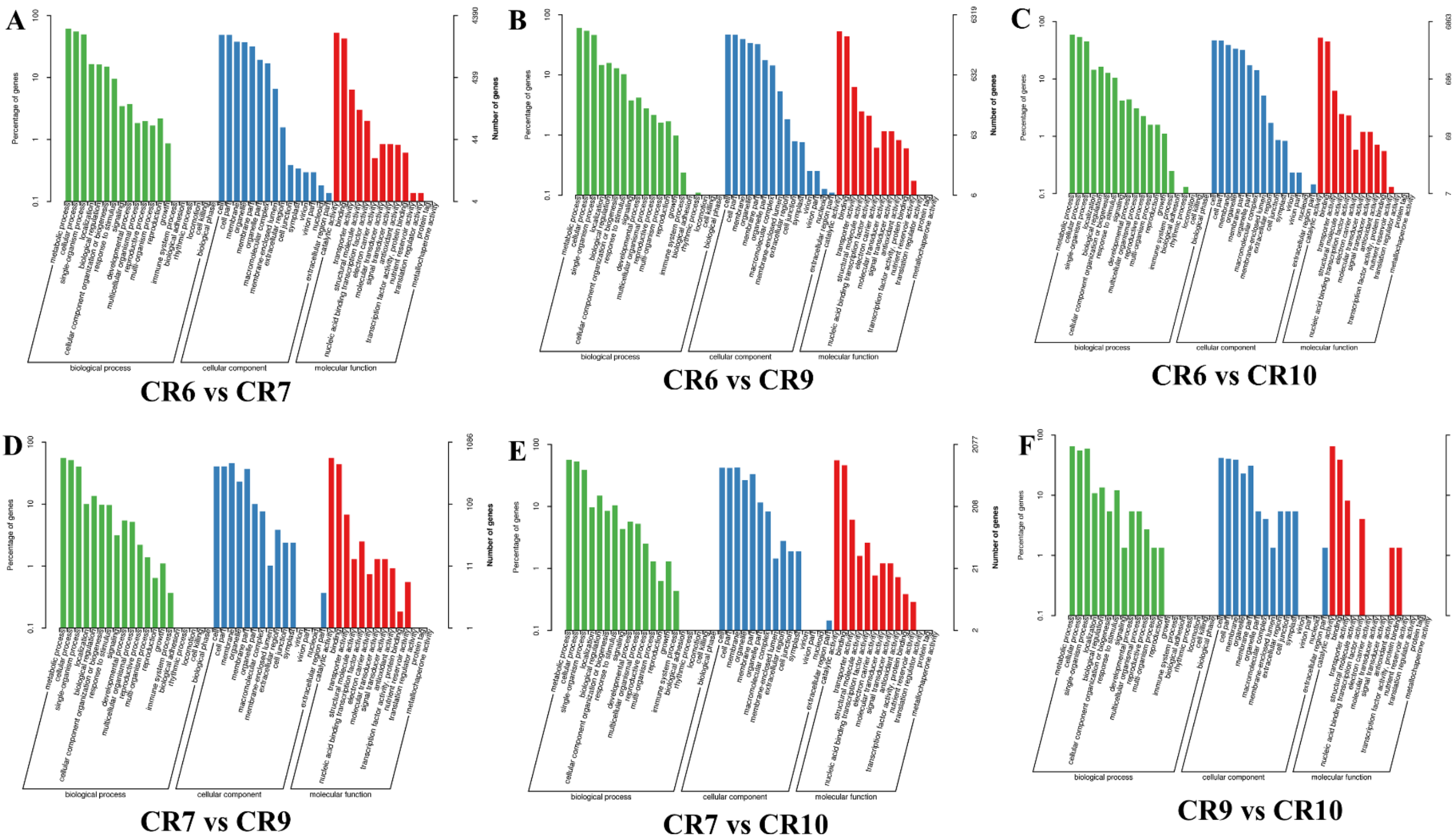

Figure supplementary 1. GO enrichment of six developmental stage comparisons. The abscissa represents the different GO term enriched genes; the ordinate represents the number of DEGs. The green bar represent DEGs enriched in biological process, the blue bar represent DEGs enriched in cellular component, the red bar represent DEGs enriched in molecular function.
